# Continuous aerobic exercise and atherosclerosis-related vascular indices in overweight and obese adults: a Bayesian three-level meta-analysis of randomized controlled trials

**DOI:** 10.3389/fphys.2026.1789752

**Published:** 2026-05-29

**Authors:** Jiang Bian, Zhiyuan Tan, Hang Yin, Jie Yang

**Affiliations:** 1 School of Arts and Sciences, Chengdu College of University of Electronic Science and Technology of China, Chengdu, China; 2 Faculty of Sport and Physical Education, University of Belgrade, Belgrade, Serbia; 3 Department of Sports Studies, Faculty of Educational Studies, Universiti Putra Malaysia, Serdang, Malaysia

**Keywords:** arterial stiffness, Bayesian three-level meta-analysis, carotid intima–media thickness, continuous aerobic exercise, continuous endurance training, endothelial function, flow-mediated dilation, obesity

## Abstract

**Background:**

Overweight and obesity accelerate vascular aging and atherosclerosis-related processes through endothelial dysfunction and increased arterial stiffness. Whether continuous endurance training (CET) improves key vascular functional and structural indices in overweight/obese adults remains uncertain, and evidence synthesis is complicated by correlated effect sizes within trials.

**Objective:**

To quantify the effects of CET on flow-mediated dilation (FMD), pulse wave velocity (PWV), and carotid intima–media thickness (CIMT) in overweight and obese adults using a Bayesian three-level meta-analysis of randomized controlled trials (RCTs).

**Methods:**

PubMed, Web of Science, Cochrane Library, Embase, APA PsycINFO, and EBSCO-SportDiscus were searched from inception to November 2025. Eligible studies were RCTs enrolling adults (18–65 years) with overweight/obesity, comparing CET with usual care/usual lifestyle, and reporting baseline and post-intervention data for FMD, PWV, or CIMT (≥4 weeks). Standardized mean differences (SMDs) for between-group changes were synthesized using a Bayesian three-level random-effects model to account for within-study dependency. Risk of bias was assessed with RoB 2, and certainty of evidence with GRADE.

**Results:**

Seventeen RCTs (746 participants) were included. CET improved FMD (13 studies, 14 effect sizes; μ = 1.14, 95% credible interval [CrI] 0.42 to 1.95), with substantial between-study heterogeneity. CET reduced PWV (7 studies, 9 effect sizes; μ = −0.23, 95% CrI −0.41 to −0.06) with minimal heterogeneity, indicating a consistent benefit on arterial stiffness. The effect on CIMT was not conclusive (5 studies, 5 effect sizes; μ = −0.23, 95% CrI −0.66 to 0.18). Exploratory subgroup analyses suggested the most consistent improvements clustered around interventions lasting 9–12 weeks and performed three sessions per week. GRADE certainty was low for FMD, moderate for PWV, and very low for CIMT, mainly due to risk of bias and imprecision.

**Conclusions:**

In overweight and obese adults, CET yields a “function-first” vascular response—improving endothelial function (FMD) and reducing arterial stiffness (PWV)—while evidence for short- to mid-term structural change in CIMT remains insufficient. Future well-designed RCTs with longer follow-up and standardized vascular assessments are needed to clarify CET effects on vascular remodeling.

**Systematic review registration:**

https://www.crd.york.ac.uk/prospero/, identifier CRD42024593701.

## 1 Introduction

Overweight and obesity have become major, modifiable health risk factors worldwide and contribute substantially to the onset and progression of cardiovascular disease (CVD) (The Global BMI Mortality Collaboration, 2016; Lopez-Jimenez et al., 2022). In obesity, insulin resistance, chronic low-grade inflammation, heightened oxidative stress, and neurohumoral dysregulation act in concert to impair endothelial function and promote unfavorable changes in arterial mechanics and structural remodeling, thereby accelerating atherosclerosis-related processes and increasing the risk of cardiovascular events (Furukawa et al., 2004; Mengozzi et al., 2020; Koenen et al., 2021; Li et al., 2021). Consequently, identifying effective and sustainable non-pharmacological strategies for adults with overweight/obesity is of considerable clinical and public health importance for reducing the burden of CVD (Jensen et al., 2014).

Compared with metabolic outcomes such as body weight or lipid profiles, vascular functional and structural indices more directly capture key pathophysiological pathways relevant to vascular aging and atherosclerotic disease and can serve as clinically meaningful intermediate endpoints along the continuum from risk factors to hard events (Townsend et al., 2015; Thijssen et al., 2019). Flow-mediated dilation (FMD) reflects endothelium-dependent vasodilatory capacity and has been associated with subsequent cardiovascular risk (Inaba et al., 2010). Pulse wave velocity (PWV) is a widely used marker of arterial stiffness with established prognostic relevance for cardiovascular outcomes and all-cause mortality (Vlachopoulos et al., 2010). Carotid intima–media thickness (CIMT) represents arterial wall remodeling and subclinical atherosclerotic burden and is similarly linked to future cardiovascular events (Lorenz et al., 2007).

Continuous endurance training (CET), as a first-line lifestyle intervention, is thought to improve vascular health through multiple, complementary mechanisms (Ashor et al., 2015; Bull et al., 2020). Exercise-induced elevations in pulsatile shear stress can activate endothelial mechanotransduction pathways, enhance endothelial nitric oxide synthase (eNOS) activity, and improve nitric oxide (NO) bioavailability, thereby supporting endothelial function. CET may also attenuate oxidative and inflammatory burden and, at the hemodynamic level, lower resting blood pressure and sympathetic tone while improving arterial compliance, collectively influencing arterial stiffness phenotypes (Hambrecht et al., 2003; Cornelissen and Smart, 2013). Nonetheless, randomized controlled trials (RCTs) conducted in adults with overweight/obesity have varied substantially in exercise prescription (duration, frequency, intensity, and adherence), participant phenotype, and vascular assessment protocols (e.g., measurement pathways and devices for PWV, imaging sites and reading standards for CIMT), which likely contributes to variability in the direction and magnitude of observed effects (Li et al., 2023).

At the same time, overweight/obesity should not be viewed as a homogeneous vascular-risk state. Vascular responsiveness to CET may differ according to obesity phenotype and baseline cardiometabolic burden, including metabolically healthy versus metabolically unhealthy obesity and the presence of hypertension, insulin resistance, dyslipidemia, or related comorbidities. This heterogeneity is clinically relevant because baseline endothelial dysfunction, arterial stiffness, and the capacity for exercise-induced adaptation may vary substantially across phenotypes, thereby contributing to between-study variability in observed vascular effects (Mengozzi et al., 2020; Li et al., 2021; Li et al., 2023).

In addition to clinical and methodological heterogeneity, evidence synthesis in this area is complicated by effect-size dependency (Moeyaert et al., 2017). In vascular intervention trials, a single RCT may report multiple correlated endpoints, multiple time points, or multiple intervention arms sharing the same control group; these effect sizes are therefore not statistically independent. Treating them as independent in conventional two-level meta-analysis can underestimate standard errors and overstate precision, potentially undermining the robustness of inference. Three-level meta-analysis addresses this problem by decomposing variability into sampling error, within-study variance, and between-study variance, thereby accounting for correlations induced by multiple effect sizes from the same study (Van den Noortgate et al., 2013). Moreover, Bayesian inference provides a transparent characterization of uncertainty through posterior distributions and can yield more stable estimation of heterogeneity parameters when study numbers are limited or heterogeneity is substantial; it also facilitates sensitivity and robustness analyzes via explicit prior specification (Röver, 2020).

Against this background and these evidence gaps, we systematically identified RCTs of CET in adults with overweight/obesity and applied a Bayesian three-level meta-analytic model to quantify the overall effects of CET on vascular functional and structural outcomes (FMD, PWV, and CIMT), while partitioning heterogeneity into within- and between-study components (Röver and Friede, 2023). We further examined key modulating factors (e.g., intervention duration and training frequency) to explore potential sources of between-study variability. We hypothesized that CET would yield more readily detectable improvements in functional and hemodynamic indices (FMD and PWV) over relatively short intervention periods, whereas effects on the structural marker CIMT might require longer exposure and greater cumulative training dose. Collectively, these findings may help inform exercise-based vascular risk management in adults with overweight/obesity, support outcome selection, and guide the design of future trials with improved methodological rigor.

## 2 Materials and methods

### 2.1 Study design and reporting guideline

This study was reported in accordance with the Preferred Reporting Items for Systematic Reviews and Meta-Analyzes (PRISMA) guideline (Appendix 1). We additionally followed relevant PRISMA extension guidance to enhance the transparency and completeness of reporting. The study protocol was prospectively registered in the International Prospective Register of Systematic Reviews (PROSPERO; registration number: CRD42024593701).

### 2.2 Information sources and search strategy

We systematically searched PubMed, Web of Science, the Cochrane Library, Embase, APA PsycINFO, and EBSCO-SportDiscus from inception to November 2025. Searches were conducted across title, abstract, and keyword fields. The search strategy was developed around the population (adults with overweight/obesity), the intervention (continuous aerobic/endurance training or aerobic exercise), and relevant outcomes (atherosclerosis, arterial stiffness, and vascular functional/structural indices), and combined using Boolean operators (“OR”/”AND”). The core search string was: (“overweight” OR “obese” OR “adult” OR “adults”) AND (“exercise” OR “aerobic exercise” OR “continuous endurance training” OR “walking” OR “running” OR “cycling” OR “endurance training”) AND (“atherosclerosis” OR “arterial stiffness” OR “carotid atherosclerosis” OR “cardiovascular health” OR “carotid intima-media thickness” OR “flow-mediated dilation” OR “pulse wave velocity”). In addition, we performed supplementary searches in Google Scholar and screened the reference lists of included studies and relevant reviews to identify potentially missed records. No publication-year restrictions were applied. We limited inclusion to English-language articles; non-English records identified during screening were documented for completeness but were not included in the quantitative synthesis.

### 2.3 Eligibility criteria

Eligibility criteria were prespecified using the PICOST framework: (1) Participants: adults (≥18 years) with overweight or obesity, as defined by the original studies, including clinically diverse cohorts in which overweight/obesity status was explicitly documented by study eligibility criteria or supported by baseline adiposity characteristics; (2) Intervention: continuous endurance training (CET), such as walking, running, or cycling, with sustained aerobic exercise as the core component; (3) Comparison: usual care/usual lifestyle without structured exercise training; (4) Outcomes: reporting baseline and post-intervention data for at least one primary outcome—flow-mediated dilation (FMD), pulse wave velocity (PWV), or carotid intima–media thickness (CIMT); (5) Study design: randomized controlled trials (RCTs); and (6) Time: intervention duration ≥4 weeks. Exclusion criteria were non-exercise interventions; participants not meeting overweight/obesity definitions according to the original study criteria or baseline adiposity characteristics; non-randomized designs; interventions not classified as CET (e.g., resistance training, interval training, or combined/multicomponent programs); ineligible outcomes or insufficient data for extraction; duplicate publications or overlapping datasets (the most informative report was retained); and unavailable full texts with key data unobtainable after attempts to contact the authors.

### 2.4 Study selection

Two reviewers independently screened the literature and cross-checked the results. All records were imported into EndNote 19 for deduplication, followed by a stepwise selection process at the title, abstract, and full-text levels to identify eligible studies. Discrepancies were resolved through discussion, and unresolved disagreements were adjudicated by a third reviewer. The study selection process is summarized in a PRISMA flow diagram, and the main reasons for full-text exclusion were documented.

### 2.5 Risk of bias assessment

Methodological quality of the included RCTs was assessed using RoB 2 in accordance with the Cochrane Handbook for Systematic Reviews of Interventions. Two reviewers independently applied the RoB 2 guidance to make domain-level judgments and cross-checked all assessments; disagreements that could not be resolved through discussion were adjudicated by a third reviewer. RoB 2 evaluates risk of bias across five prespecified domains: bias arising from the randomization process, bias due to deviations from intended interventions, bias due to missing outcome data, bias in measurement of the outcome, and bias in selection of the reported result. Each domain judgment was informed by the RoB 2 signaling questions, and an overall risk-of-bias rating was assigned to each study (low risk, some concerns, or high risk) to support a transparent and reproducible assessment of internal validity.

### 2.6 Statistical analysis

We performed the quantitative synthesis in R (version 4.5.2) using the brms package (Stan backend; Carpenter et al., 2017; Burkner, 2017) and a Bayesian three-level random-effects meta-analysis to accommodate effect-size dependency arising from multiple estimates reported within the same trial (e.g., multiple vascular outcomes, repeated time points, or multi-arm designs sharing a common control group). Rather than treating such comparisons as statistically independent, the three-level model explicitly partitioned total variability into sampling variance (level 1), within-study heterogeneity (level 2), and between-study heterogeneity (level 3), thereby accounting for non-independence while retaining all eligible effect sizes.

#### 2.6.1 Effect size calculation

Given differences in measurement scales/units across studies, we used standardized mean differences (SMDs) as the common effect measure and synthesized standardized between-group differences in change. Specifically, we first calculated pre–post changes (Δ) within each arm and then computed the between-group difference in change:


$$y\;=\;\left(\Delta \bar{X}I\right)\;-\;\left(\Delta \bar{X}C\right)$$

SMDs and their standard errors (sij) were derived and used as model inputs. When the standard deviation of change was not reported, it was derived from baseline and follow-up standard deviations using an assumed pre–post correlation (r); sensitivity analyses were conducted by varying r to assess the impact of this assumption.

#### 2.6.2 Bayesian three-level model specification

At the observation level, we assumed yij ~ Normal(θij, sij²), where sij denotes the standard error of the effect size. True effects were modeled with a three-level structure:


θij = μ + ui + vij,

where μ is the overall mean effect, ui is the between-study random effect, and vij represents within-study random effects. This specification allows multiple effect sizes from the same study to share a study-level random component, thereby explicitly modeling dependence while retaining all available information and yielding more reliable posterior uncertainty estimates.

#### 2.6.3 Priors, model fitting, and diagnostics

We used weakly informative priors: μ ~ Normal(0, 10), and τwithin and τbetween ~ half-Cauchy(0, 0.5). Models were fitted in R using brms (Stan backend) with Hamiltonian Monte Carlo (HMC). Sampling variances were incorporated via standard errors (yi | se(sei)), and the random-effects structure was specified as (1 | study_id) + (1 | study_id:effect_id), where study_id identifies the trial and effect_id indexes effect sizes within a trial. We ran four chains with 4,000 iterations per chain (2,000 warm-up iterations) and set adapt_delta = 0.99 and max_treedepth = 15 to reduce divergent transitions and improve numerical stability. Convergence was assessed using R-hat (<1.01) and effective sample size (ESS), supplemented by trace-plot inspection. Model fit was evaluated using posterior predictive checks. Pooled effects are reported as posterior means with 95% credible intervals (95% CrI) and visualized using forest plots and posterior distributions of heterogeneity parameters (τwithin and τbetween).

#### 2.6.4 Exploring heterogeneity: exploratory subgroup analyses and meta-regression

To explore potential sources of heterogeneity and generate hypotheses, we conducted exploratory moderator analyzes. Continuous prescription characteristics (e.g., intervention duration in weeks and training frequency in sessions/week) were examined using Bayesian three-level meta-regression, while categorical moderators (e.g., region) were evaluated through exploratory subgroup comparisons. Because the number of studies was limited for some outcomes, these analyzes were interpreted as exploratory and intended to aid understanding of between-study variability rather than support definitive causal inference. Meta-regression results are presented as posterior mean regression coefficients with 95% CrIs and interpreted alongside posterior predictive distributions.

Exercise intensity was also considered during data extraction; however, intensity was reported inconsistently across studies using non-equivalent prescription metrics, including percentage of maximal heart rate, heart rate reserve, percentage of peak oxygen uptake, workload targets, speed-based prescriptions, and perceived exertion. Moreover, the included CET modalities were heterogeneous, and a given nominal intensity may not reflect an equivalent hemodynamic or metabolic stimulus across exercise modes. Because the number of studies within directly comparable intensity categories was limited, a formal pooled intensity analysis was not considered sufficiently robust and was therefore not performed.

#### 2.6.5 Robustness: leave-one-out and additional sensitivity analyses

Robustness was examined using prespecified sensitivity analyses. We first conducted leave-one-out analyzes by repeating the synthesis after omitting each study in turn to assess the influence of individual studies on the pooled effect. Additional sensitivity analyses included retaining only the primary follow-up time point per study; restricting multi-arm trials to the primary CET comparison; excluding studies judged to be at high overall risk of bias or those relying on digitized extraction for key outcome data; and evaluating prior/parameter sensitivity by replacing half-Cauchy heterogeneity priors with half-normal priors and by varying the assumed pre–post correlation (r) used to derive change-score standard deviations.

#### 2.6.6 Publication bias and small-study effects

For outcomes with a sufficient number of studies, we further assessed small-study effects and potential publication bias as supplementary analyzes using funnel plots. To satisfy the independence assumption and avoid distortion from multiple effect sizes per study, publication-bias analyzes were conducted using one effect size per study; when the number of studies was insufficient for reliable testing, results were presented descriptively and interpreted cautiously.

### 2.7 Certainty of evidence

We assessed the certainty of evidence for the primary outcomes (FMD, PWV, and CIMT) using the GRADE framework. Certainty ratings were determined by considering five domains—risk of bias, inconsistency, indirectness, imprecision, and publication bias—and were categorized as high, moderate, low, or very low. These ratings were used to support the strength of conclusions and to inform the interpretation of clinical and practical implications.

## 3 Results

### 3.1 Study selection

**Figure 1:**
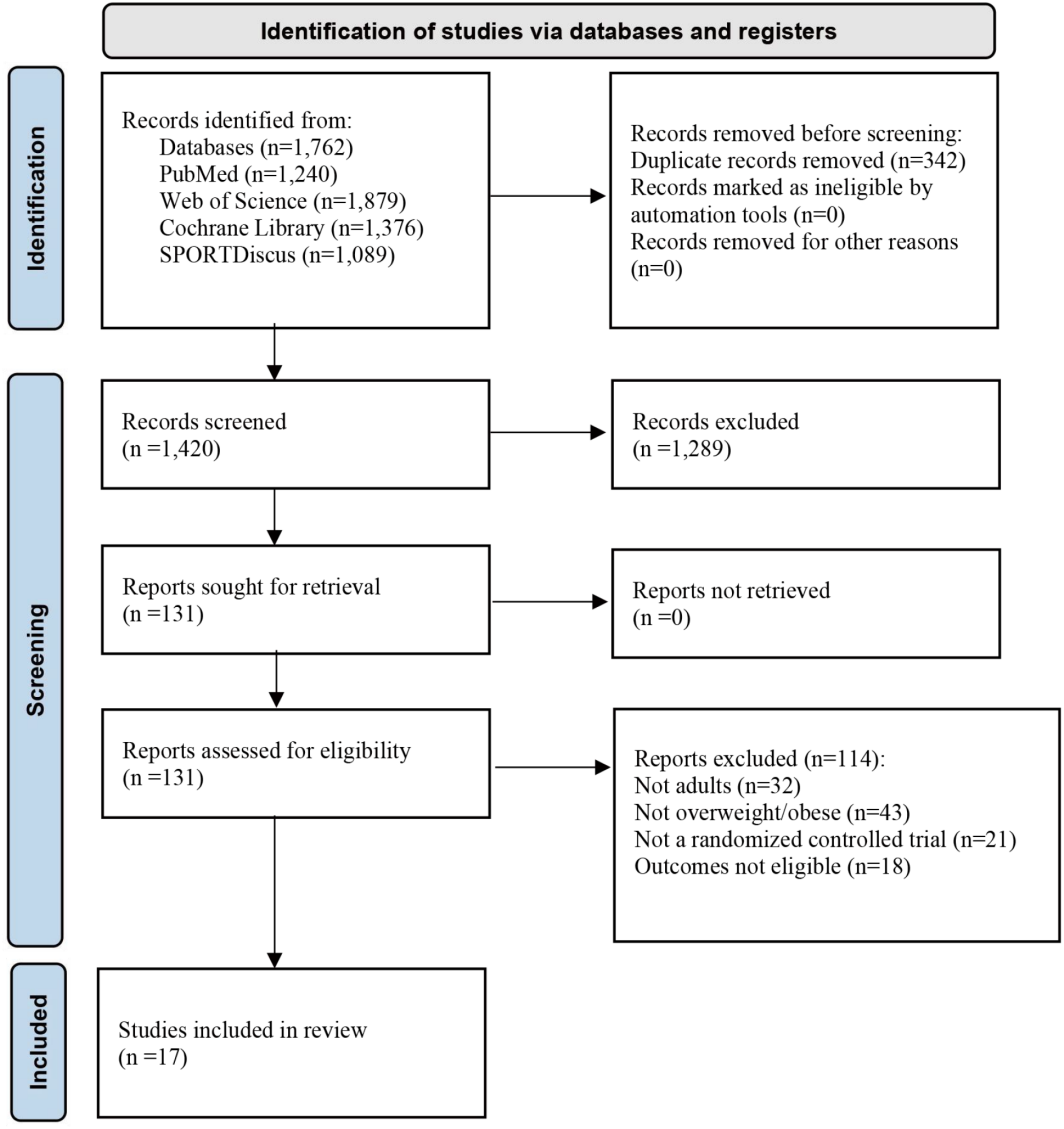
Flow diagram of study selection for trials included in the systematic review.

A total of 1,762 records were retrieved from six databases. After importing the results into EndNote 19 and removing 342 duplicates, 1,420 records remained. Following stepwise screening of titles and abstracts, 131 articles were retained for full-text review after excluding non-exercise interventions and studies irrelevant to the topic. After full-text assessment, 114 articles were excluded because the participants were not adult populations, overweight/obesity status was not supported by the original study criteria or baseline adiposity characteristics, the study was not a randomized controlled trial, or the outcomes did not meet the inclusion criteria. Together with additional studies identified through other sources, 17 studies were finally included (Bircher and Mucha, 2007; Braith et al., 2008; Nualnim et al., 2012; Donley et al., 2014; Headley et al., 2014; Pugh et al., 2014; Greenwood et al., 2015; Oliveira et al., 2015; Van Craenenbroeck et al., 2015; Robinson et al., 2016; Azadpour et al., 2017; Rahbar et al., 2018; Slivovskaja et al., 2018; Kirkman et al., 2019; Gholami et al., 2020; Jo et al., 2020; He et al., 2022). The study selection process is shown in **Figure 1**.

### 3.2 Study quality assessment

**Figure 2:**
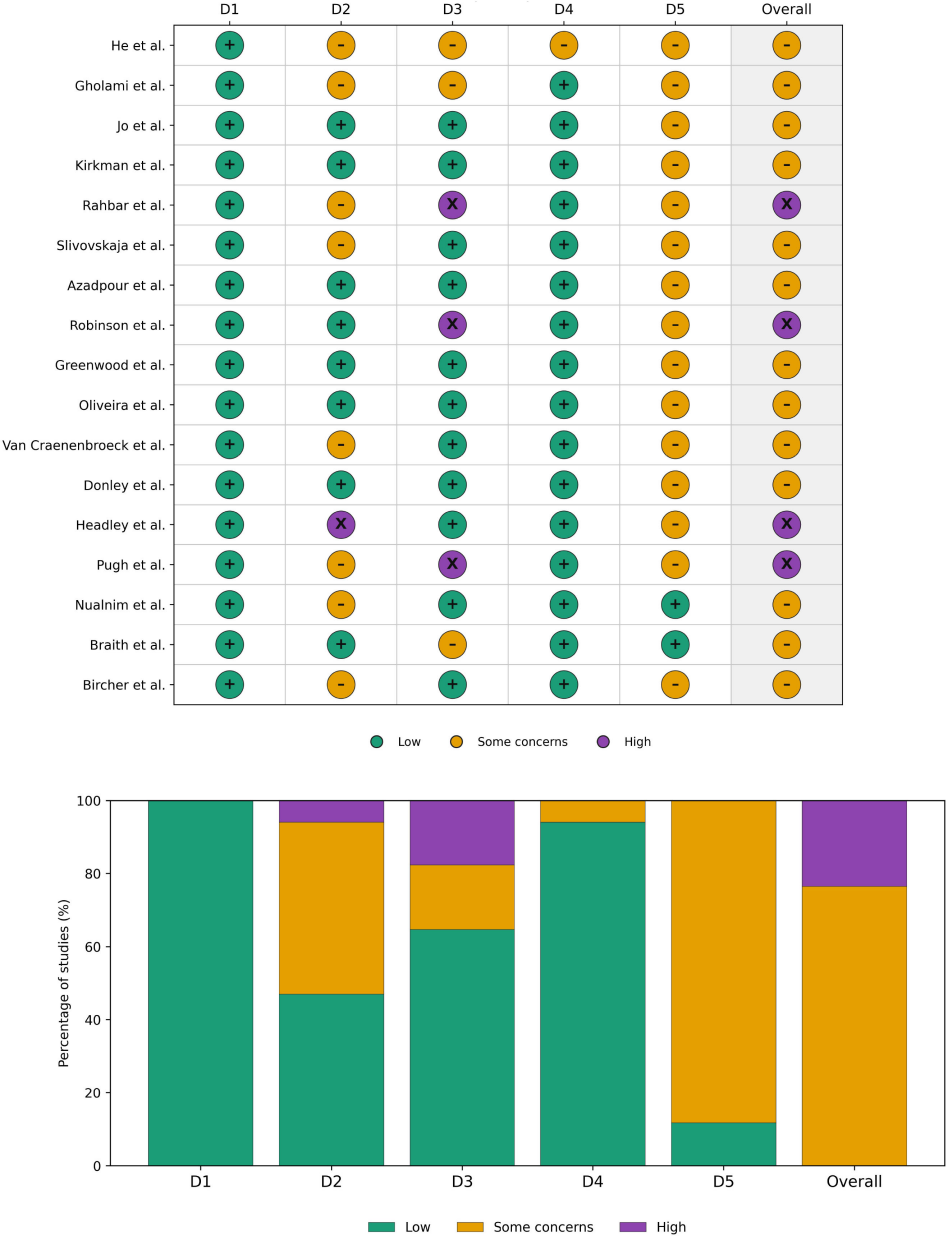
Quality evaluation of each study. D1, randomization process; D2, deviations from intended interventions; D3, missing outcome data; D4, measurement of the outcome; D5, selection of the reported result; D6, overall.

Overall, the RoB 2 assessment indicated that most included trials raised some concerns (13/17), with 4/17 judged at high risk of bias (**Figure 2**). All studies were rated low risk for the randomization process (D1: 17/17). Concerns clustered in selective reporting (D5: 15/17 some concerns) and deviations from intended interventions (D2: 8/17 some concerns; 1/17 high risk), while high risk due to missing outcome data (D3) was identified in 3/17 trials. Collectively, randomization appeared adequate, whereas reporting transparency, intervention implementation, and missing-data handling may reduce confidence in the evidence.

### 3.3 Basic characteristics of the included studies

The basic characteristics of the 17 included randomized controlled trials are summarized in **Table 1**. A total of 746 overweight/obese adults were included (393 in the intervention group and 353 in the control group) and were randomly allocated to intervention or control conditions. All studies applied traditional continuous endurance training (CET), with intervention durations ranging from 8 to 16 weeks and training frequencies of 3–5 sessions per week. Pugh et al. was a three-arm trial and He et al. was a two-arm trial, while the remaining studies included a single intervention arm; control groups generally maintained usual lifestyle/usual care. The main outcomes included FMD, PWV, and/or CIMT.

**Table 1:** Basic information of the included studies.

Study	Countries	RCT arms	n (intervention)	BMI (intervention)	n (control)	BMI (control)	Intervention characteristics			Main outcomes	Participant characteristics
							Type	Length	Frequency		Clinical phenotype
Azadpour et al.(2017) (Azadpour et al., 2017)	Istanbul	1	12	>30	12	>30	CET	10	3	FMD	Obese postmenopausal women with prehypertension
Jo et al.(2020) (Jo et al., 2020)	South Korea	1	13	27.0 ± 3.0	13	27.3 ± 4.6	CET	12	4	FMD	Postmenopausal women with high cardiovascular risk
Donley et al.(2014) (Donley et al., 2014)	United States	1	11	34 ± 2	11	38 ± 2	CET	8	3	PWV、CIMT	Adults with metabolic syndrome
Slivovskaja et al.(2018) (Slivovskaja et al., 2018)	Lithuania	1	84	27.3 ± 4.6	42	27.0 ± 4.7	CET	8	5	PWV、CIMT	Adults with metabolic syndrome
Kirkman et al.(2019) (Kirkman et al., 2019)	United States	1	15	27.1 ± 4.4	16	27.1 ± 4.4	CET	12	3	PWV、FMD	Adults with nondialysis chronic kidney disease
Rahbar et al.(2018) (Rahbar et al., 2018)	Iran	1	13	28 ± 1	15	28 ± 2	CET	8	3	CIMT	Adults with diabetes
Oliveira et al.(2015) (Oliveira et al., 2015)	Portugal	1	37	27 ± 2.1	41	26 ± 3.3	CET	8	3	PWV	Patients with myocardial infarction undergoing cardiac rehabilitation
Nualnim et al.(2012) (Nualnim et al., 2012)	United States	1	24	28 ± 3.3	19	28± 4.3	CET	12	3	PWV、FMD	Adults >50 years of age
Robinson et al.(2016) (Robinson et al., 2016)	United States	1	10	28.3 ± 4	9	28.3 ± 4.1	CET	8	3	FMD	Overweight and obese adults
Greenwood et al.(2015) (Greenwood et al., 2015)	United Kingdom	1	13	30± 1.1	20	30 ± 1.2	CET	12	3	PWV	Kidney transplant recipients
Pugh et al.(2014) (Pugh et al., 2014)	United Kingdom	3	13	27.3 ± 4.6	8	27.3 ± 4.6	CET	16	3	FMD	Adults with nonalcoholic fatty liver disease
			13	27.3 ± 4.6	15	27.3 ± 4.6	CET	16	3	FMD	Adults with nonalcoholic fatty liver disease
			23	27.3 ± 4.6	25	27.3 ± 4.6	CET	16	3	FMD	Adults with nonalcoholic fatty liver disease
Van Craenenbroeck et al.(2015) (Van Craenenbroeck et al., 2015)	Belgium	1	19	28.7± 3.3	21	28.7± 3.3	CET	12	4	FMD	Adults with CKD stages 3-4
Braith et al.(2008) (Braith et al., 2008)	United States	1	9	29 ± 3	7	30 ± 1	CET	12	3	FMD	Heart transplant recipients
He et al.(2022) (He et al., 2022)	China	2	15	25± 4.6	15	25± 4.6	CET	8	5	FMD	Postmenopausal females
			15	25± 4.6	15	25± 4.6	CET	8	3	FMD	Postmenopausal females
Headley et al.(2014) (Headley et al., 2014)	United States	1	25	29 ± 1	21	29 ± 2	CET	16	3	PWV	Adults with CKD stage 3
Gholami et al.(2020) (Gholami et al., 2020)	Iran	1	16	32 ± 1.1	15	32 ± 2.1	CET	12	3	FMD、CIMT	Adults with type 2 diabetes and peripheral neuropathy
Bircher et al.(2007) (Bircher and Mucha, 2007)	Germany	1	13	26.9± 2.7	13	27.3 ± 1.4	CET	12	3	FMD	Obese adults

CET, continuous endurance training; FMD, flow-mediated dilation; PWV, pulse wave velocity; CIMT, carotid intima–media thickness; CKD, chronic kidney disease. Participant characteristics/clinical phenotype were extracted from the original trial descriptions. When detailed baseline cardiometabolic risk factors were not uniformly reported, only the main clinical phenotype or comorbidity of the study population is shown.

In addition to differences in exercise prescription, the included trials represented heterogeneous cardiometabolic phenotypes. Several studies enrolled overweight/obese adults with coexisting conditions such as prehypertension, metabolic syndrome, chronic kidney disease, nonalcoholic fatty liver disease, or type 2 diabetes, whereas detailed reporting of hypertension status, insulin resistance, lipid abnormalities, and other baseline risk factors was inconsistent in other trials. This incomplete phenotypic characterization should be considered when interpreting pooled vascular responses.

### 3.4 Primary outcome findings

**Figure 3:**
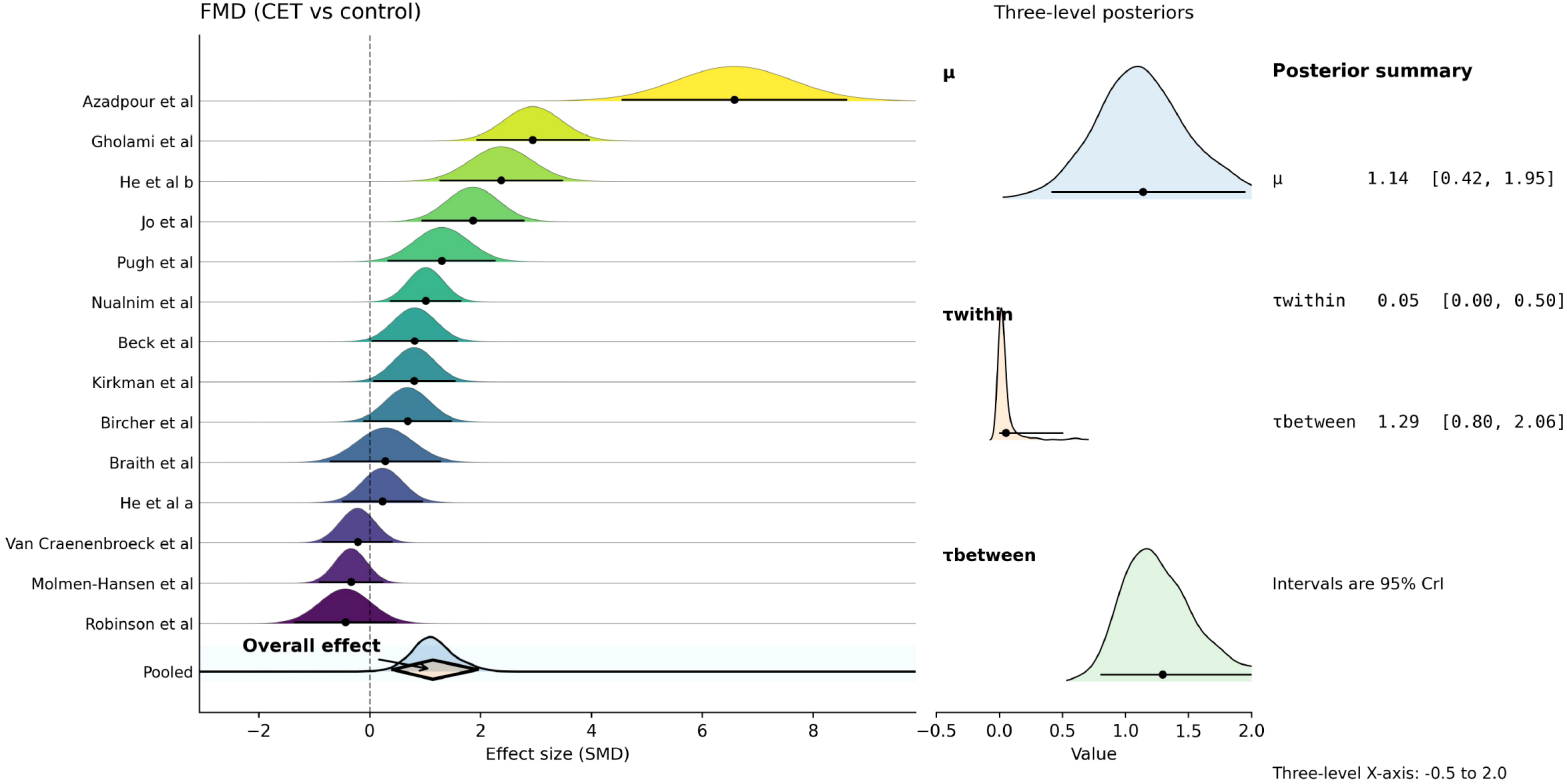
Bayesian three-level random-effects meta-analysis of CET versus control on FMD. Left: study-specific SMDs, standardized mean differences and pooled estimate. Right: posterior distributions for the overall mean effect (μ) and heterogeneity components (τ_within and τ_between); bars indicate 95% CrI, credible intervals.

Thirteen studies contributing 14 effect sizes were included in the analysis of FMD (**Figure 3**). Because outcome metrics and units varied across trials, effects were pooled as standardized mean differences (SMDs) using a Bayesian three-level random-effects model comparing CET with control. The pooled mean effect for FMD was μ = 1.14 (95% credible interval [CrI], 0.42 to 1.95). Between-study heterogeneity was substantial (τbetween = 1.29; 95% CrI, 0.80 to 2.06), whereas within-study heterogeneity was small (τwithin = 0.05; 95% CrI, 0.00 to 0.50).

**Figure 4:**
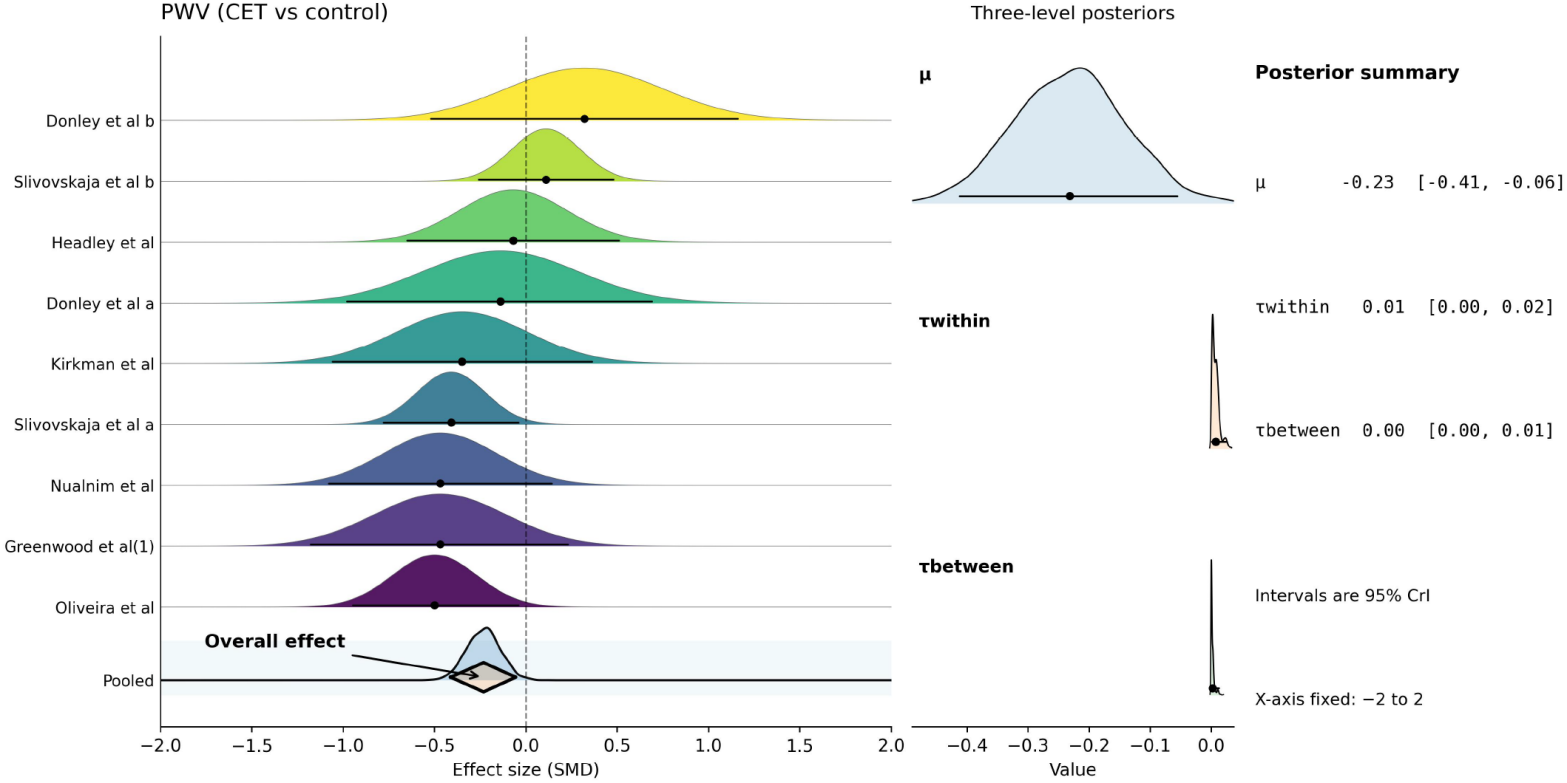
Bayesian three-level random-effects meta-analysis of CET versus control on PWV. Left: study-specific SMDs, standardized mean differences and pooled estimate. Right: posterior distributions for the overall mean effect (μ) and heterogeneity components (τ_within and τ_between); bars indicate 95% CrI, credible intervals.

Seven studies contributing nine effect sizes were included in the analysis of PWV (**Figure 4**). Effects were synthesized as SMDs using the same Bayesian three-level random-effects framework. The pooled mean effect for PWV was μ = −0.23 (95% CrI, −0.41 to −0.06). Heterogeneity was minimal at both levels, with τbetween = 0.00 (95% CrI, 0.00 to 0.01) and τwithin = 0.01 (95% CrI, 0.00 to 0.02).

**Figure 5:**
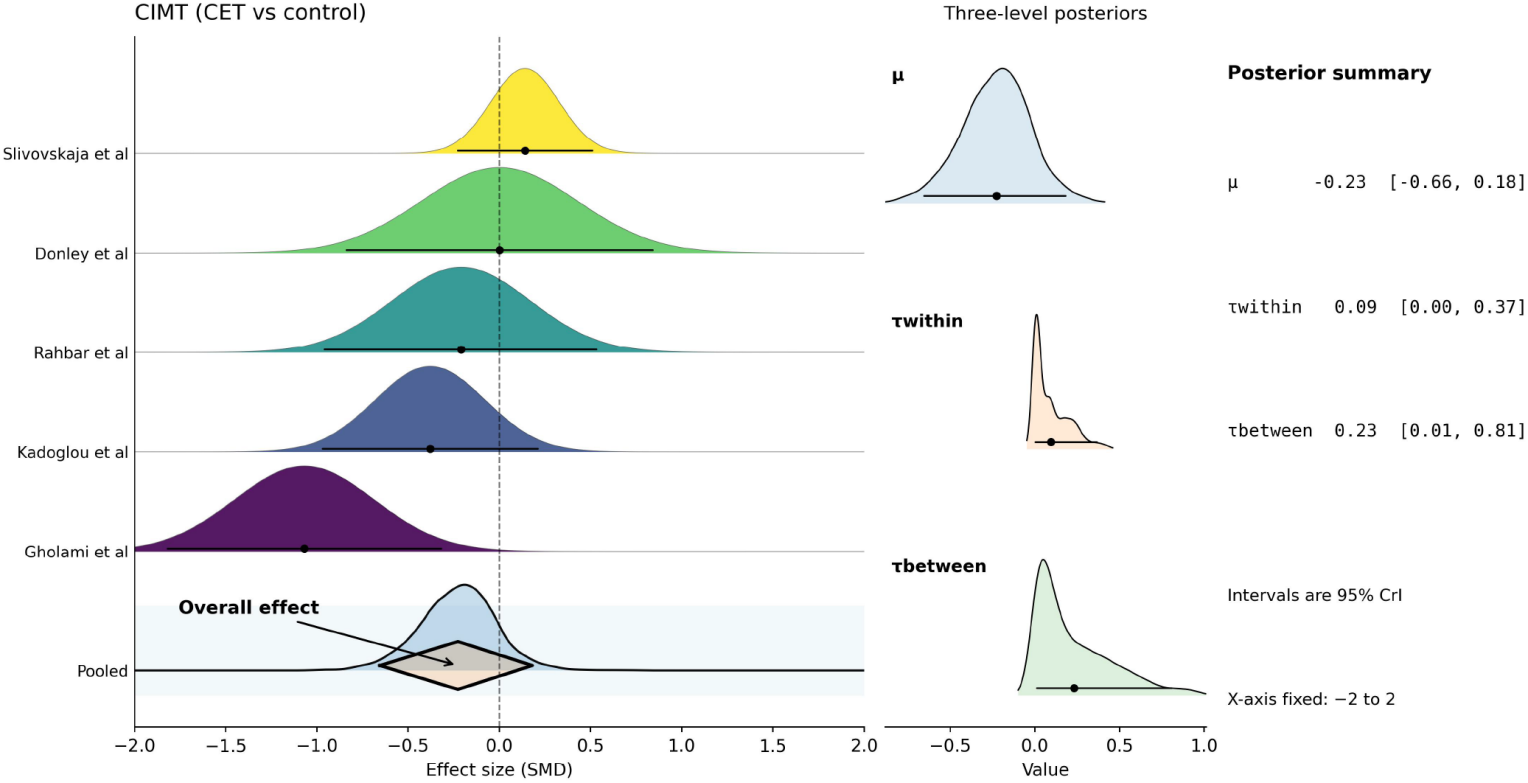
Bayesian three-level random-effects meta-analysis of CET versus control on CIMT Left: study-specific SMDs, standardized mean differences and pooled estimate. Right: posterior distributions for the overall mean effect (μ) and heterogeneity components (τ_within and τ_between); bars indicate 95% CrI, credible intervals.

Five studies contributing five effect sizes were included in the analysis of CIMT (**Figure 5**). The pooled mean effect for CIMT was μ = −0.23 (95% CrI, −0.66 to 0.18). Between-study heterogeneity was modest (τbetween = 0.23; 95% CrI, 0.01 to 0.81), and within-study heterogeneity was also modest (τwithin = 0.09; 95% CrI, 0.00 to 0.37). Because the 95% CrI included 0, the pooled CIMT result was not statistically conclusive.

### 3.5 Model diagnostics

**Figure 6:**
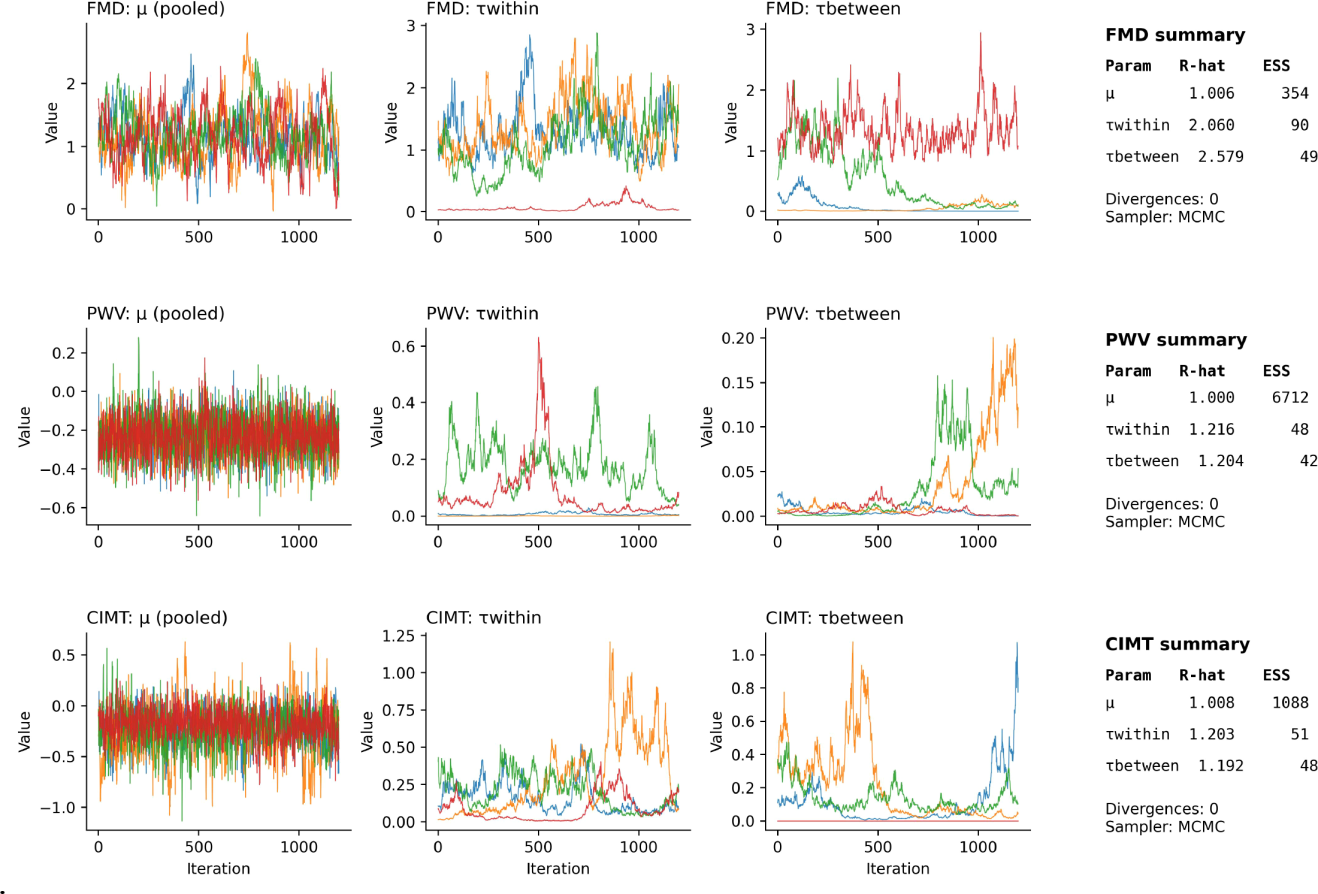
MCMC diagnostics for the Bayesian three-level meta-analytic models. Trace plots are shown for the pooled mean effect (μ), within-study heterogeneity (τ_within), and between-study heterogeneity (τ_between) for FMD, PWV, and CIMT. Summary statistics for each parameter, including R-hat and ESS, effective sample size, are presented on the right; divergences indicate the number of divergent transitions during sampling.

**Figure 7:**
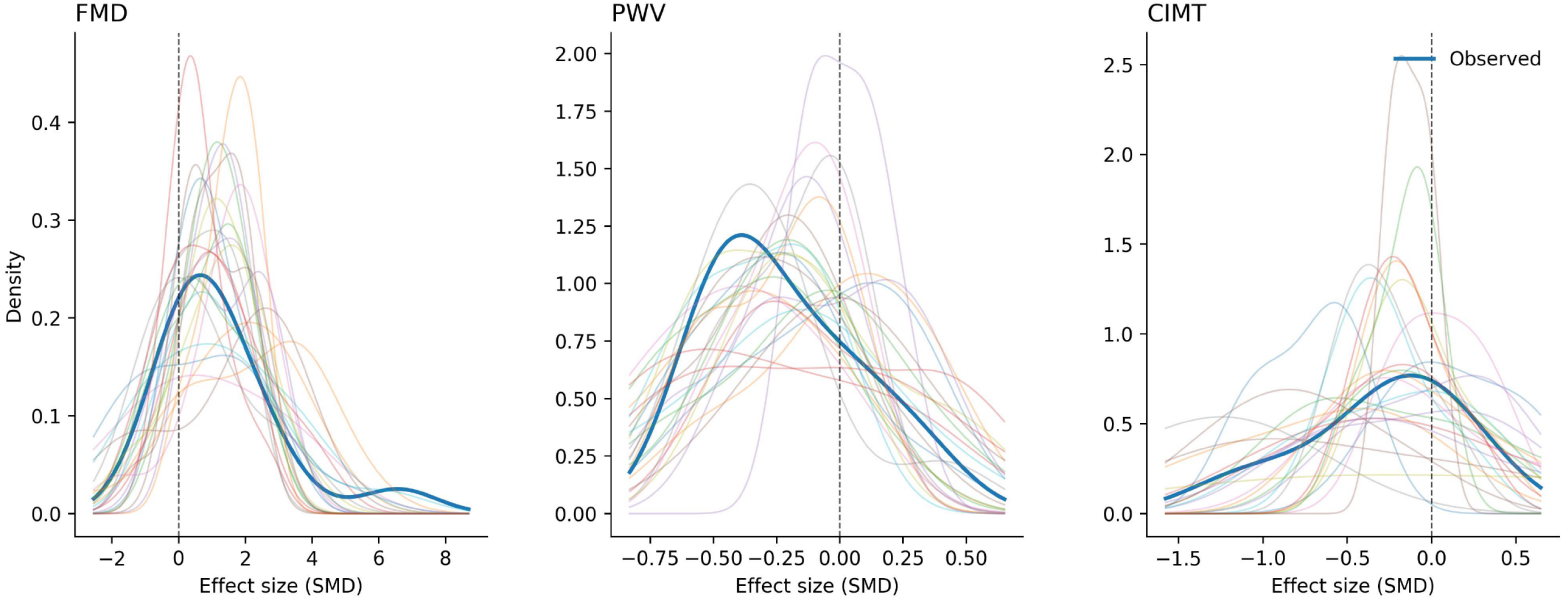
Posterior predictive checks for the Bayesian three-level meta-analytic models. For each outcome (FMD, PWV, and CIMT), the observed effect-size density is shown as a thick blue curve, whereas posterior predictive densities simulated from the fitted model are shown as colored thin curves; dashed vertical lines indicate the null effect (SMD = 0).

Model diagnostics are presented in **Figures 6** and **7**. Across outcomes, no divergent transitions were observed, indicating stable numerical sampling. Convergence of the pooled mean effect (μ) was acceptable for FMD (R-hat = 1.006; ESS = 354), PWV (R-hat = 1.000; ESS = 6712), and CIMT (R-hat = 1.008; ESS = 1088). In contrast, convergence for heterogeneity parameters was weaker. For FMD, τwithin and τbetween showed elevated R-hat values (2.060 and 2.579, respectively) and low ESS values (90 and 49). For PWV, τwithin and τbetween showed R-hat values of 1.216 and 1.204 with ESS values of 48 and 42. For CIMT, τwithin and τbetween showed R-hat values of 1.203 and 1.192 with ESS values of 51 and 48. Posterior predictive checks showed that the simulated posterior distributions broadly covered the observed effect-size distributions for all three outcomes. Model fit appeared strongest for PWV. For FMD, predictive coverage was broader in the right tail, whereas for CIMT the predictive densities were more dispersed, indicating greater posterior uncertainty for that outcome.

### 3.6 Exploratory subgroup and meta-regression analyzes

Exploratory subgroup analyses by intervention duration, weekly frequency, and region are presented in **Table 2**. For FMD, subgroup estimates were 0.699 (95% CI, −0.301 to 1.698) for interventions ≤8 weeks, 1.273 (95% CI, 0.425 to 2.121) for 9–12 weeks, and 1.300 (95% CI, 0.335 to 2.265) for >12 weeks. By frequency, subgroup estimates were 1.249 (95% CI, 0.514 to 1.985) for 3 sessions/week and 0.580 (95% CI, −0.546 to 1.705) for ≥4 sessions/week. Regional estimates were 2.610 (95% CI, 1.044 to 4.176) for Asia, 0.476 (95% CI, −0.148 to 1.101) for the Americas, and 0.529 (95% CI, −0.353 to 1.411) for Europe. For PWV, subgroup estimates were −0.182 (95% CI, −0.485 to 0.121) for interventions ≤8 weeks, −0.434 (95% CI, −0.821 to −0.048) for 9–12 weeks, and −0.070 (95% CI, −0.650 to 0.510) for >12 weeks. By frequency, subgroup estimates were −0.304 (95% CI, −0.544 to −0.064) for 3 sessions/week and −0.150 (95% CI, −0.660 to 0.360) for ≥4 sessions/week. Regional estimates were −0.182 (95% CI, −0.491 to 0.126) for the Americas and −0.283 (95% CI, −0.598 to 0.031) for Europe. For CIMT, subgroup estimates were 0.061 (95% CI, −0.247 to 0.369) for interventions ≤8 weeks, −1.070 (95% CI, −1.820 to −0.320) for 9–12 weeks, and −0.380 (95% CI, −0.970 to 0.210) for >12 weeks. By frequency, subgroup estimates were −0.442 (95% CI, −1.086 to 0.203) for 3 sessions/week and −0.067 (95% CI, −0.566 to 0.432) for ≥4 sessions/week. Regional estimates were −0.639 (95% CI, −1.482 to 0.204) for Asia, 0.000 (95% CI, −0.840 to 0.840) for the Americas, and 0.140 (95% CI, −0.230 to 0.510) for Europe.

**Table 2:** Subgroup analyses of vascular outcomes following CET.

Indicator	Moderator	Subgroup	k	RE SMD	95% CI	I² (%)	τ²
FMD							
Duration subgroup							
FMD	Duration	≤8 weeks	4	0.699	[-0.301, 1.698]	81.5	0.839
FMD	Duration	9–12 weeks	9	1.273	[0.425, 2.121]	90.0	1.453
FMD	Duration	>12 weeks	1	1.300	[0.335, 2.265]	0.0	0.000
Frequency subgroup							
FMD	Frequency	3/week	11	1.249	[0.514, 1.985]	87.7	1.306
FMD	Frequency	≥4/week	3	0.580	[-0.546, 1.705]	85.3	0.839
Region subgroup							
FMD	Region	Asia	6	2.610	[1.044, 4.176]	91.2	2.822
FMD	Region	Americas	5	0.476	[-0.148, 1.101]	58.8	0.236
FMD	Region	Europe	4	0.529	[-0.353, 1.411]	73.6	0.444
CIMT							
Duration subgroup							
CIMT	Duration	≤8 weeks	3	0.061	[-0.247, 0.369]	0.0	0.000
CIMT	Duration	9–12 weeks	1	-1.070	[-1.820, -0.320]	0.0	0.000
CIMT	Duration	>12 weeks	1	-0.380	[-0.970, 0.210]	0.0	0.000
Frequency subgroup							
CIMT	Frequency	3/week	3	-0.442	[-1.086, 0.203]	51.5	0.167
CIMT	Frequency	≥4/week	2	-0.067	[-0.566, 0.432]	53.3	0.072
Region subgroup							
CIMT	Region	Asia	3	-0.639	[-1.482, 0.204]	60.7	0.224
CIMT	Region	Americas	1	0.000	[-0.840, 0.840]	0.0	0.000
CIMT	Region	Europe	1	0.140	[-0.230, 0.510]	0.0	0.000
PWV							
Duration subgroup							
PWV	Duration	≤8 weeks	5	-0.182	[-0.485, 0.121]	43.6	0.049
PWV	Duration	9–12 weeks	3	-0.434	[-0.821, -0.048]	0.0	0.000
PWV	Duration	>12 weeks	1	-0.070	[-0.650, 0.510]	0.0	0.000
Frequency subgroup							
PWV	Frequency	3/week	7	-0.304	[-0.544, -0.064]	0.0	0.000
PWV	Frequency	≥4/week	2	-0.150	[-0.660, 0.360]	73.6	0.100
Region subgroup							
PWV	Region	Americas	5	-0.182	[-0.491, 0.126]	0.0	0.000
PWV	Region	Europe	4	-0.283	[-0.598, 0.031]	49.0	0.049

CET, continuous endurance training; FMD, flow-mediated dilation; PWV, pulse wave velocity; CIMT, carotid intima–media thickness; k, number of effect sizes/study comparisons contributing to each subgroup; RE SMD, random-effects standardized mean difference; CI, confidence interval; I², proportion of total variability attributable to between-study heterogeneity; τ², estimated between-study variance. These subgroup analyses were exploratory and are presented for descriptive comparison of potential heterogeneity patterns. Accordingly, they should not be interpreted as definitive exercise prescription thresholds.

**Figure 8:**
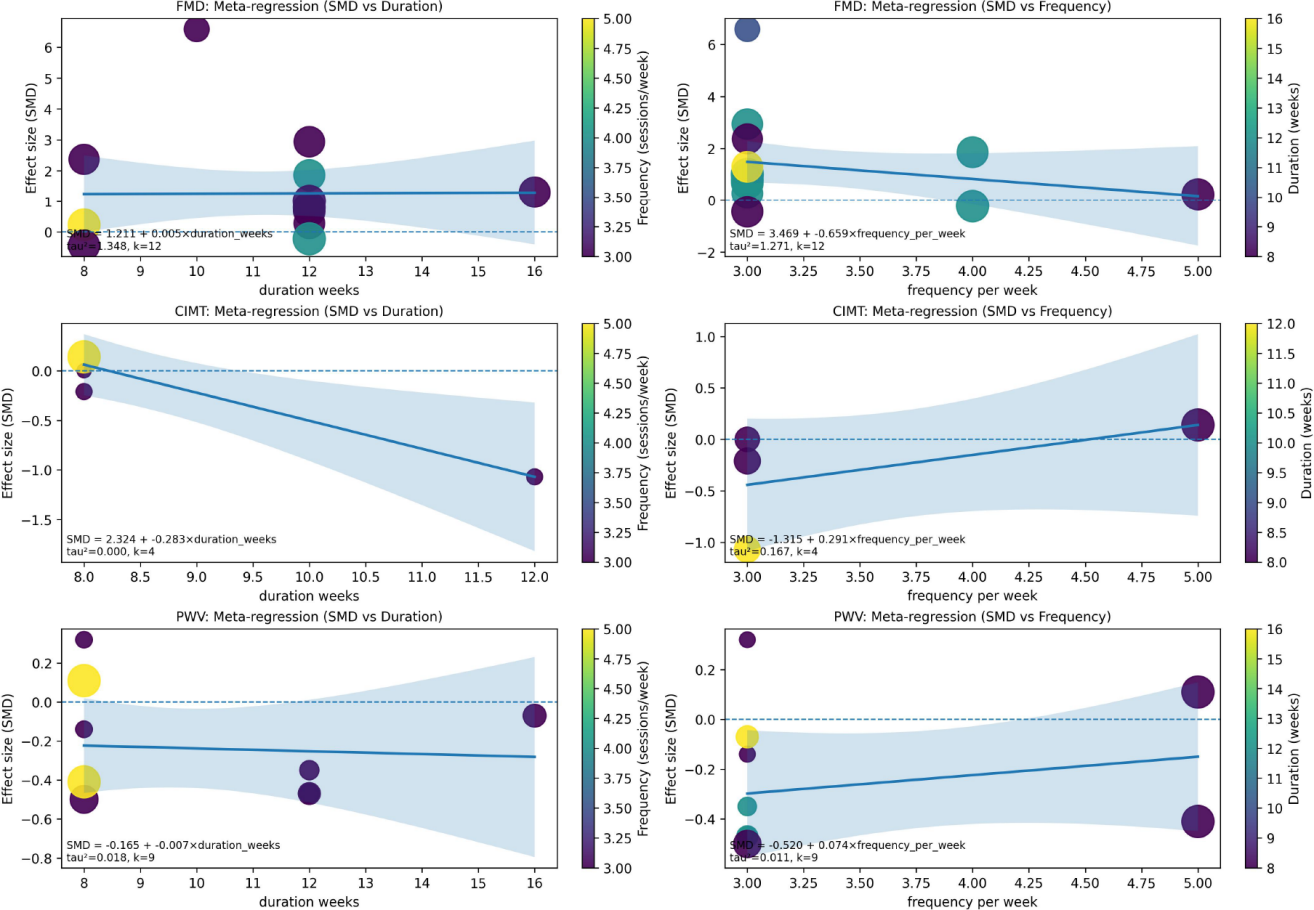
Exploratory meta-regression analyses of CET prescription characteristics and vascular outcomes. Panels show study-level SMDs, standardized mean differences plotted against intervention duration (weeks) and training frequency (sessions/week) for FMD, CIMT, and PWV. Solid blue lines indicate the estimated meta-regression slopes, and shaded areas indicate 95% credible intervals. Points represent study-level effect sizes; color scales indicate the alternate prescription characteristic (frequency for duration models and duration for frequency models).

Exploratory meta-regression analyses are shown in **Figure 8**. For FMD, the estimated slope for intervention duration was approximately +0.003 per week, whereas the slope for training frequency was approximately −0.659 per additional session/week. For CIMT, the estimated slope for duration was approximately −0.283 per week and for frequency approximately +0.291 per additional session/week. For PWV, estimated slopes were approximately −0.007 per week for duration and +0.074 per additional session/week for frequency. Confidence bands were wide across models.

### 3.7 Sensitivity analyses and publication bias

**Figure 9:**
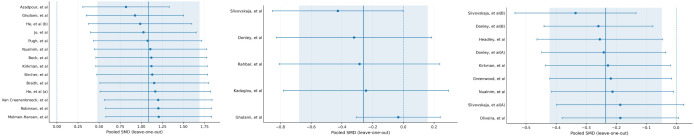
Leave-one-out sensitivity analyses for the Bayesian three-level meta-analytic models. For each outcome (FMD, PWV, and CIMT), the pooled effect estimate was recalculated after omitting one study at a time to evaluate the influence of individual trials on the overall result.

**Figure 10:**
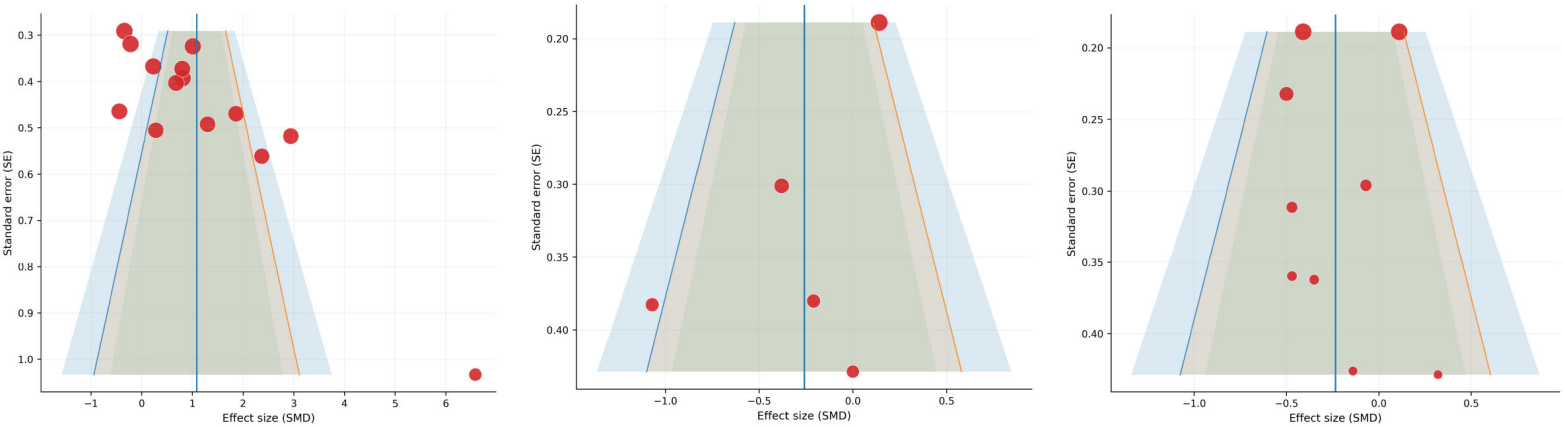
Funnel plots for assessment of small-study effects and potential publication bias. Separate funnel plots are shown for FMD, PWV, and CIMT. Each point represents one study-level effect size plotted against its standard error; the vertical line indicates the pooled effect estimate, and diagonal lines indicate pseudo 95% confidence limits.

Building on the primary analyses, we conducted leave-one-out sensitivity analyses (**Figure 9**) to identify potentially influential studies and determine whether the overall conclusions were driven by any single trial. Overall, the direction of effects for improved FMD and reduced PWV remained stable across iterations, whereas inference for CIMT remained constrained by limited sample size and imprecision, underscoring the need for additional high-quality studies. Funnel plots (**Figure 10**) were used to assess potential publication bias and small-study effects. The panels correspond to the FMD, PWV, and CIMT outcomes, respectively. Most studies were concentrated toward the top of the plots and, as standard errors decreased, effect estimates converged toward the center, yielding an overall symmetric inverted-funnel pattern, which suggests a low likelihood of publication bias. No obvious anomalies or outlying points were observed, providing additional support for the robustness of the findings.

### 3.8 Certainty of evidence

**Table 3** presents the GRADE-based assessment of the certainty of evidence for vascular outcomes after continuous endurance training (CET) in overweight/obese adults (SMD, 95% CrI). CET may improve FMD [1.14 (0.42 to 1.95)] with low certainty and reduce PWV [-0.23 (-0.41 to -0.06)] with moderate certainty, whereas the effect on CIMT remains uncertain [-0.23 (-0.66 to 0.18)] with very low certainty, mainly due to risk of bias and imprecision.

**Table 3:** GRADE assessment of the certainty of evidence for vascular outcomes following CET.

Outcome	Studies	Participants	Effect (SMD, 95% CrI)	RoB	Inconsistency	Indirectness	Imprecision	Publication bias	Certainty
FMD	13 (14)	Overweight/ obese adults	1.14 (0.42 to 1.95)	Serious¹	Serious²	Not serious	Serious³	Not detected⁴	Low
PWV	7 (9)	Overweight/ obese adults	-0.23 (-0.41 to -0.06)	Serious¹	Not serious	Not serious	Serious³	Not detected⁴	Moderate
cIMT	5 (5)	Overweight/ obese adults	-0.23 (-0.66 to 0.18)	Serious¹	Serious²	Not serious	Very serious³	Unable to assess⁴	Very low

CET, continuous endurance training; FMD, flow-mediated dilation; PWV, pulse wave velocity; CIMT, carotid intima–media thickness; SMD, standardized mean difference; CrI, credible interval; RoB, risk of bias. Certainty of evidence was assessed using the GRADE framework across the domains of risk of bias, inconsistency, indirectness, imprecision, and publication bias.

## 4 Discussion

This study synthesized randomized controlled trial (RCT) evidence in adults with overweight/obesity and quantitatively pooled vascular outcomes of continuous endurance training (CET) using a Bayesian three-level random-effects model. The findings support a clear “function-first” pattern: CET improved flow-mediated dilation (FMD) and reduced pulse wave velocity (PWV), indicating concordant improvements in endothelial function and arterial stiffness phenotypes after training, whereas the pooled effect on carotid intima–media thickness (CIMT) was not yet conclusive, suggesting insufficient evidence for structural reversal over the short to medium term. Clinically, these results imply that, in adults with overweight/obesity, CET is more likely to yield earlier, observable functional benefits (enhanced endothelial reactivity and reduced stiffness) rather than stable, short-term changes in vessel wall structure (Hambrecht et al., 2003; Laurent et al., 2006; Seravalle and Grassi, 2016; Grassi et al., 2019; Saxton et al., 2019; Thijssen et al., 2019). For clinical management and trial design, if the objective is to capture short- to mid-term improvements in risk-related vascular phenotypes, FMD and PWV are more suitable as primary endpoints (Seravalle and Grassi, 2016; Grassi et al., 2019; Thijssen et al., 2019). If CIMT is selected as a core endpoint, longer follow-up, greater cumulative training dose, and more rigorous imaging standardization should be anticipated; otherwise, conclusions will be more vulnerable to limited precision and measurement heterogeneity (Guzik et al., 2000; Hambrecht et al., 2003).

From a pathophysiological perspective, vascular injury associated with overweight/obesity is typically driven by multiple interacting pathways, including insulin resistance, chronic low-grade inflammation, heightened oxidative stress, perivascular adipose tissue (PVAT) dysfunction, and increased sympathetic activity (Tinken et al., 2010; Lorenz et al., 2012; Touboul et al., 2012; Green et al., 2014; Green et al., 2017; Bakali et al., 2023). Accordingly, the improvements in FMD and PWV observed with CET likely reflect an “early plasticity response” arising from coordinated, multi-target effects (Laurent et al., 2006; Vlachopoulos et al., 2010; Ashor et al., 2015; Thijssen et al., 2019). Hemodynamically, repeated exercise-induced increases in blood flow elevate vascular shear stress, activating endothelial mechanotransduction and promoting eNOS activation, thereby enhancing nitric oxide (NO)-mediated vasodilation—changes that can manifest as measurable FMD improvements within the short to medium term (Laurent et al., 2006; Vlachopoulos et al., 2010; Ashor et al., 2015; Thijssen et al., 2019; Hejazi and Wong, 2023). In obesity, NO bioavailability is often constrained by increased oxidative NO scavenging and eNOS uncoupling (Touboul et al., 2012; Tayebi et al., 2017; Saeidi et al., 2019b); thus, the net training effect plausibly depends on concurrent gains at both ends of the pathway—augmenting NO production while limiting NO inactivation (Ashor et al., 2015; Tayebi et al., 2017; Saeidi et al., 2019b; Saeidi et al., 2020). In parallel, attenuation of inflammatory and oxidative stress states may further reduce endothelial activation and vascular tone load, providing a coherent biological basis for both improved FMD and decreased PWV (Touboul et al., 2012; Saeidi et al., 2019a; Saeidi et al., 2023). Prior studies indicate that aerobic training can reduce inflammatory markers such as hs-CRP, IL-6, and TNF-α, and may lower selected adhesion/endothelial activation markers (e.g., sICAM-1, sVCAM-1, E-selectin), thereby improving the endothelial milieu under inflammatory conditions (Saeidi et al., 2019a; Hejazi and Wong, 2023; Saeidi et al., 2023; Zouhal et al., 2023). Exercise may also influence L-arginine–related metabolic pathways and NO-generating processes, increasing the likelihood of NO pathway recovery (Saeidi et al., 2019b; Saeidi et al., 2020). Moreover, training-induced antioxidant and mitochondrial adaptations (including upregulation of antioxidant enzyme systems and activation of transcriptional networks related to mitochondrial biogenesis) may reduce reactive oxygen species (ROS) burden and NO inactivation, increasing the “visibility” of training effects on endothelium-dependent vasodilation (Ashor et al., 2014; Tourny et al., 2023). Collectively, these mechanisms are consistent with the notion that, in adults with overweight/obesity, CET preferentially improves vascular functional status (FMD, PWV) first, whereas structural indices (CIMT) may require a longer time window and stronger cumulative stimuli to change reliably (Guzik et al., 2000; Hambrecht et al., 2003).

Importantly, dose (duration/frequency) and regional subgrouping in this study should be interpreted primarily as tools to explore heterogeneity and generate hypotheses, rather than as substitutes for causal inference from the main analysis (Viechtbauer, 2010; Van den Noortgate et al., 2013; Van den Noortgate et al., 2015; Burkner, 2017). We observed relatively consistent benefit signals clustering within a prescription window of 9–12 weeks with three sessions per week; in regional stratification, significant FMD benefits were more readily detected in Asian studies, whereas PWV and CIMT did not show stable regional differences. Interpretation of “regional effects” should remain conservative: such patterns more likely reflect differences in baseline risk profiles, intervention implementation intensity and adherence, and measurement protocols (devices, measurement paths, reading standards) rather than a biological causal effect of region per se (Hambrecht et al., 2003; Seravalle and Grassi, 2016; Thijssen et al., 2019). Likewise, across the broader evidence base, PWV is not uniformly moderated by prescription components; some foundational work suggests that more pronounced improvements may relate to longer-term vascular wall adaptation (“arterial remodeling”), with a plausible temporal sequence of “functional change preceding structural remodeling”—early shifts dominated by endothelial reactivity and vascular tone regulation, followed by slower structural adaptations (Ashor et al., 2015; Seravalle and Grassi, 2016; Grassi et al., 2019; Saxton et al., 2019). Under resting conditions, vessel diameter may not expand substantially, potentially due to compensatory adjustments in constrictor/dilator tone that alter shear exposure and thereby facilitate endothelial functional improvement (Vlachopoulos et al., 2010; Ashor et al., 2015).

This evidence structure—clearer overall effects but more cautious interpretation of subgroup signals—is consistent with our modeling strategy and diagnostic findings. The key rationale for adopting a Bayesian three-level random-effects model was to address effect-size dependency: individual RCTs frequently include multiple intervention arms sharing a control group, multiple outcomes, and/or multiple time-point reports, meaning effect sizes are not statistically independent; treating them as independent in conventional two-level models can underestimate standard errors and inflate apparent precision (Viechtbauer, 2010; Van den Noortgate et al., 2013; Van den Noortgate et al., 2015; Burkner, 2017). The three-level framework decomposes total variability into sampling error, within-study variability, and between-study variability, explicitly representing within-study correlations at the model level and thereby strengthening the robustness of pooled estimates (Viechtbauer, 2010; Van den Noortgate et al., 2015; Burkner, 2017). Because Bayesian summaries (μ and τ parameters) are derived from MCMC posterior sampling, we assessed computational reliability using convergence diagnostics and posterior predictive checks (PPC), and used leave-one-out sensitivity analyses to identify potentially influential studies (Gelman and Rubin, 1992; Vehtari et al., 2017). Overall, these checks provide stronger support for interpreting μ (the direction and magnitude of the overall effect), making the directional conclusions for FMD and PWV relatively more robust; by contrast, uncertainty around CIMT appears primarily constrained by the number of studies and limitations in measurement precision and standardization (Guzik et al., 2000; Hambrecht et al., 2003).

### 4.1 Limitations of the review

It should be noted that this Bayesian three-level meta-analysis has several potential limitations. First, with respect to eligibility, all included studies were randomized controlled trials (RCTs) using continuous aerobic exercise interventions; however, such trials cannot be fully blinded. As a result, subjective factors may have introduced bias into study conduct and quality appraisal. Second, substantial between-study heterogeneity was observed, which may be attributable to multiple vascular risk factors and co-exposures, including smoking, diet and alcohol intake, blood pressure, and lipid profiles. Third, because the evidence base for CIMT was limited, we were unable to adequately account for inter-individual variability or perform more granular, personalized subgroup analyses for this structural endpoint. Finally, given the marked heterogeneity in the pooled estimates, the conclusions of this meta-analysis should be interpreted with appropriate caution. In addition, exercise intensity was not analyzed quantitatively because prescription and reporting were not sufficiently standardized across studies, limiting our ability to evaluate its contribution to between-study heterogeneity.

### 4.2 Conclusion

Our analysis indicates that continuous aerobic exercise improves flow-mediated dilation (FMD) and reduces pulse wave velocity (PWV) in adults with overweight/obesity. We further observed that improvements across these vascular indices were related to exercise prescription characteristics, particularly intervention duration and training frequency. Specifically, the most consistent benefit signals clustered within a 9–12-week program delivered three times per week. In contrast, evidence for structural benefit on carotid intima–media thickness (CIMT) remains insufficient, underscoring the need for higher-quality trials with longer follow-up to determine whether CET can reliably induce vascular wall remodeling.

## Data Availability

The original contributions presented in the study are included in the article/supplementary material. Further inquiries can be directed to the corresponding author.

## References

[B1] Ashor A. W. Lara J. Siervo M. Celis-Morales C. Mathers J. C. (2014). Effects of exercise modalities on arterial stiffness and wave reflection: a systematic review and meta-analysis of randomized controlled trials. PloS One 9, e110034. doi: 10.1371/journal.pone.0110034. PMID: 25333969 PMC4198209

[B2] Ashor A. W. Lara J. Siervo M. Celis-Morales C. Oggioni C. Jakovljevic D. G. . (2015). Exercise modalities and endothelial function: a systematic review and dose-response meta-analysis of randomized controlled trials. Sports Med. 45, 279–296. doi: 10.1007/s40279-014-0272-9. PMID: 25281334

[B3] Azadpour N. Tartibian B. Koşar Ş. (2017). Effects of aerobic exercise training on ACE and ADRB2 gene expression, plasma angiotensin II level, and flow-mediated dilation: a study on obese postmenopausal women with prehypertension. Menopause 24, 269–277. doi: 10.1097/GME.0000000000000762. PMID: 28231078

[B4] Bakali M. Ward T. C. J. Daynes E. Jones A. V. Hawthorne G. M. Latimer L. . (2023). Effect of aerobic exercise training on pulse wave velocity in adults with and without long-term conditions: a systematic review and meta-analysis. Open Heart 10, e002384. doi: 10.1136/openhrt-2023-002384. PMID: 38101857 PMC10729135

[B5] Bircher S. Mucha C. (2007). Einfluss körperlicher Aktivität auf die Fettoxidation und Endothelfunktion bei Adipösen – eine randomisiert kontrollierte Studie. Phys. Med. Rehabil. Kurortmed 17, 313–319. doi: 10.1055/s-2007-973832. PMID: 3785596

[B6] Braith R. W. Schofield R. S. Hill J. A. Casey D. P. Pierce G. L. (2008). Exercise training attenuates progressive decline in brachial artery reactivity in heart transplant recipients. J. Heart Lung Transplant. 27, 52–59. doi: 10.1016/j.healun.2007.09.032. PMID: 18187087

[B7] Bull F. C. Al-Ansari S. S. Biddle S. Borodulin K. Buman M. P. Cardon G. . (2020). World Health Organization 2020 guidelines on physical activity and sedentary behaviour. Br. J. Sports Med. 54, 1451–1462. doi: 10.1136/bjsports-2020-102955. PMID: 33239350 PMC7719906

[B8] Burkner P. C. (2017). brms: an R package for Bayesian multilevel models using Stan. J. Stat. Softw 80, 1–28. doi: 10.18637/jss.v080.i01

[B9] Carpenter B. Gelman A. Hoffman M. D. Lee D. Goodrich B. Betancourt M. . (2017). Stan: a probabilistic programming language. J. Stat. Softw 76, 1–32. doi: 10.18637/jss.v076.i01. PMID: 36568334 PMC9788645

[B10] Cornelissen V. A. Smart N. A. (2013). Exercise training for blood pressure: a systematic review and meta-analysis. J. Am. Heart Assoc. 2, e004473. doi: 10.1161/JAHA.112.004473. PMID: 23525435 PMC3603230

[B11] Donley D. A. Fournier S. B. Reger B. L. DeVallance E. Bonner D. E. Olfert I. M. . (2014). Aerobic exercise training reduces arterial stiffness in metabolic syndrome. J. Appl. Physiol. (1985) 116, 1396–1404. doi: 10.1152/japplphysiol.00151.2014. PMID: 24744384 PMC4044399

[B12] Furukawa S. Fujita T. Shimabukuro M. Iwaki M. Yamada Y. Nakajima Y. . (2004). Increased oxidative stress in obesity and its impact on metabolic syndrome. J. Clin. Invest. 114, 1752–1761. doi: 10.1172/JCI21625. PMID: 15599400 PMC535065

[B13] Gelman A. Rubin D. B. (1992). Inference from iterative simulation using multiple sequences. Stat. Sci. 7, 457–472. doi: 10.1214/ss/1177011136

[B14] Gholami F. Nazari H. Alimi M. (2020). Cycle training improves vascular function and neuropathic symptoms in patients with type 2 diabetes and peripheral neuropathy: a randomized controlled trial. Exp. Gerontol 131, 110799. doi: 10.1016/j.exger.2019.110799. PMID: 31899340

[B15] Grassi G. Biffi A. Seravalle G. Trevano F. Q. Dell’Oro R. Corrao G. . (2019). Sympathetic neural overdrive in the obese and overweight state: a meta-analysis of published studies. Hypertension 74, 349–358. doi: 10.1161/HYPERTENSIONAHA.119.12885. PMID: 31203727

[B16] Green D. J. Dawson E. A. Groenewoud H. M. M. Jones H. Thijssen D. H. J. (2014). Is flow-mediated dilation nitric oxide mediated?: a meta-analysis. Hypertension 63, 376–382. doi: 10.1161/HYPERTENSIONAHA.113.02044. PMID: 24277765

[B17] Green D. J. Hopman M. T. E. Padilla J. Laughlin M. H. Thijssen D. H. J. (2017). Vascular adaptation to exercise in humans: role of hemodynamic stimuli. Physiol. Rev. 97, 495–528. doi: 10.1152/physrev.00014.2016. PMID: 28151424 PMC5539408

[B18] Greenwood S. A. Koufaki P. Mercer T. H. Rush R. O’Connor E. Tuffnell R. . (2015). Aerobic or resistance training and pulse wave velocity in kidney transplant recipients: a 12-week pilot randomized controlled trial (the Exercise in Renal Transplant Trial). Am. J. Kidney Dis. 66, 689–698. doi: 10.1053/j.ajkd.2015.06.016. PMID: 26209542

[B19] Guzik T. J. West N. E. J. Black E. McDonald D. Ratnatunga C. Pillai R. . (2000). Vascular superoxide production by NAD(P)H oxidase: association with endothelial dysfunction and clinical risk factors. Circ. Res. 86, e85–e90. doi: 10.1161/01.RES.86.9.e85. PMID: 10807876

[B20] Hambrecht R. Adams V. Erbs S. Linke A. Kränkel N. Shu Y. . (2003). Regular physical activity improves endothelial function in patients with coronary artery disease by increasing phosphorylation of endothelial nitric oxide synthase. Circulation 107, 3152–3158. doi: 10.1161/01.CIR.0000074229.93804.5C. PMID: 12810615

[B21] He H. Wang C. Chen X. Sun X. Wang Y. Yang J. . (2022). The effects of HIIT compared to MICT on endothelial function and hemodynamics in postmenopausal females. J. Sci. Med. Sport 25, 364–371. doi: 10.1016/j.jsams.2022.01.007. PMID: 35210180

[B22] Headley S. Germain M. Wood R. Joubert J. Milch C. Evans E. . (2014). Short-term aerobic exercise and vascular function in CKD stage 3: a randomized controlled trial. Am. J. Kidney Dis. 64, 222–229. doi: 10.1053/j.ajkd.2014.02.022. PMID: 24776325 PMC4112010

[B23] Hejazi K. Wong A. (2023). Effects of exercise training on inflammatory and cardiometabolic health markers in overweight and obese adults: a systematic review and meta-analysis of randomized controlled trials. J. Sports Med. Phys. Fitness 63, 345–359. doi: 10.23736/S0022-4707.22.14103-4 35816146

[B24] Inaba Y. Chen J. A. Bergmann S. R. (2010). Prediction of future cardiovascular outcomes by flow-mediated vasodilatation of brachial artery: a meta-analysis. Int. J. Cardiovasc. Imaging 26, 631–640. doi: 10.1007/s10554-010-9616-1. PMID: 20339920

[B25] Jensen M. D. Ryan D. H. Apovian C. M. Ard J. D. Comuzzie A. G. Donato K. A. . (2014). 2013 AHA/ACC/TOS guideline for the management of overweight and obesity in adults. Circulation 129, S102–S138. doi: 10.1161/01.cir.0000437739.71477.ee. PMID: 24222017 PMC5819889

[B26] Jo E. Wu S. Han H. Park J. Park S. Cho K. (2020). Effects of exergaming in postmenopausal women with high cardiovascular risk: a randomized controlled trial. Clin. Cardiol. 43, 363–370. doi: 10.1002/clc.23324. PMID: 31883278 PMC7144488

[B27] Kirkman D. L. Ramick M. G. Muth B. J. Stock J. M. Pohlig R. T. Townsend R. R. . (2019). Effects of aerobic exercise on vascular function in nondialysis chronic kidney disease: a randomized controlled trial. Am. J. Physiol. Renal Physiol. 316, F898–F905. doi: 10.1152/ajprenal.00539.2018. PMID: 30810061 PMC6580257

[B28] Koenen M. Hill M. A. Cohen P. Sowers J. R. (2021). Obesity, adipose tissue and vascular dysfunction. Circ. Res. 128, 951–968. doi: 10.1161/CIRCRESAHA.121.318093. PMID: 33793327 PMC8026272

[B29] Laurent S. Cockcroft J. Van Bortel L. Boutouyrie P. Giannattasio C. Hayoz D. . (2006). Expert consensus document on arterial stiffness: methodological issues and clinical applications. Eur. Heart J. 27, 2588–2605. doi: 10.1093/eurheartj/ehl254. PMID: 17000623

[B30] Li M. Qian M. Kyler K. Xu J. (2021). Adipose tissue-endothelial cell interactions in obesity-induced endothelial dysfunction. Front. Cardiovasc. Med. 8, 681581. doi: 10.3389/fcvm.2021.681581 34277732 PMC8282205

[B31] Li P. Liu Z. Wan K. Wang K. Zheng C. Huang J. (2023). Effects of regular aerobic exercise on vascular function in overweight or obese older adults: a systematic review and meta-analysis. J. Exerc Sci. Fit 21, 313–325. doi: 10.1016/j.jesf.2023.06.002. PMID: 37520931 PMC10372915

[B32] Lopez-Jimenez F. Almahmeed W. Bays H. Cuevas A. Di Angelantonio E. le Roux C. W. . (2022). Obesity and cardiovascular disease: mechanistic insights and management strategies. A joint position paper by the World Heart Federation and World Obesity Federation. Eur. J. Prev. Cardiol. 29, 2218–2237. doi: 10.1093/eurjpc/zwac187 36007112

[B33] Lorenz M. W. Polak J. F. Kavousi M. Mathiesen E. B. Völzke H. Tuomainen T. P. . (2012). Carotid intima-media thickness progression to predict cardiovascular events: a meta-analysis. Lancet 379, 2053–2062. doi: 10.1016/S0140-6736(12)60441-3. PMID: 22541275 PMC3918517

[B34] Lorenz M. W. Markus H. S. Bots M. L. Rosvall M. Sitzer M. (2007). Prediction of clinical cardiovascular events with carotid intima-media thickness: a systematic review and meta-analysis. Circulation 115, 459–467. doi: 10.1161/CIRCULATIONAHA.106.628875. PMID: 17242284

[B35] Mengozzi A. Masi S. Virdis A. (2020). Obesity-related endothelial dysfunction: moving from classical to emerging mechanisms. Endocr. Metab. Sci. 1, 100063. doi: 10.1016/j.endmts.2020.100063. PMID: 38826717

[B36] Moeyaert M. Ugille M. Beretvas S. N. Ferron J. Bunuan R. Van den Noortgate W. (2017). Methods for dealing with multiple outcomes in meta-analysis: comparing averaging, robust variance estimation and multilevel meta-analysis. Int. J. Soc Res. Methodol. doi: 10.1080/13645579.2016.1252189. PMID: 37339054

[B37] Nualnim N. Parkhurst K. Dhindsa M. Tarumi T. Vavrek J. Tanaka H. (2012). Effects of swimming training on blood pressure and vascular function in adults >50 years of age. Am. J. Cardiol. 109, 1005–1010. doi: 10.1016/j.amjcard.2011.11.029. PMID: 22244035

[B38] Oliveira N. L. Ribeiro F. Silva G. Alves A. J. Silva N. Guimarães J. T. . (2015). Effect of exercise-based cardiac rehabilitation on arterial stiffness and inflammatory and endothelial dysfunction biomarkers: a randomized controlled trial of myocardial infarction patients. Atherosclerosis 239, 150–157. doi: 10.1016/j.atherosclerosis.2014.12.057. PMID: 25602857

[B39] Pugh C. J. A. Sprung V. S. Kemp G. J. Richardson P. Shojaee-Moradie F. Umpleby A. M. . (2014). Exercise training reverses endothelial dysfunction in nonalcoholic fatty liver disease. Am. J. Physiol. Heart Circ. Physiol. 307, H1298–H1306. doi: 10.1152/ajpheart.00306.2014. PMID: 25193471

[B40] Rahbar S. Naimi S. S. RezaSoltani A. Rahimi A. Baghban A. A. Noori A. . (2018). Changes in vascular structure in diabetic patients after 8 weeks aerobic physical exercise: a randomized controlled trial. Int. J. Diabetes Dev. Ctries. 38, 202–208. doi: 10.1007/s13410-017-0579-9. PMID: 30311153

[B41] Robinson A. T. Franklin N. C. Norkeviciute E. Bian J. T. Babana J. C. Szczurek M. R. . (2016). Improved arterial flow-mediated dilation after exertion involves hydrogen peroxide in overweight and obese adults following aerobic exercise training. J. Hypertens. 34, 1309–1316. doi: 10.1097/HJH.0000000000000946. PMID: 27137176

[B42] Röver C. (2020). Bayesian random-effects meta-analysis using the bayesmeta R package. J. Stat. Softw 93, 1–51. doi: 10.18637/jss.v093.i06

[B43] Röver C. Friede T . (2023). Using the bayesmeta R package for Bayesian random-effects meta-regression. Comput. Methods Programs Biomed. 229, 107303. doi: 10.1016/j.cmpb.2022.107303 36566650

[B44] Saeidi A. Jabbour G. Ahmadian M. Abbassi-Daloii A. Malekian F. Hackney A. C. . (2019a). Independent and combined effects of antioxidant supplementation and circuit resistance training on selected adipokines in postmenopausal women. Front. Physiol. 10, 484. doi: 10.3389/fphys.2019.00484. PMID: 31105587 PMC6499001

[B45] Saeidi A. Saei M. A. Mohammadi B. Akbarzadeh Zarei H. R. Vafaei M. Mohammadi A. S. . (2023). Supplementation with spinach-derived thylakoid augments the benefits of high intensity training on adipokines, insulin resistance and lipid profiles in males with obesity. Front. Endocrinol. (Lausanne) 14, 1141796. doi: 10.3389/fendo.2023.1141796. PMID: 37576981 PMC10422041

[B46] Saeidi A. Seifi-Ski-Shahr F. Soltani M. Daraei A. Shirvani H. Laher I. . (2020). Resistance training, gremlin 1 and macrophage migration inhibitory factor in obese men: a randomised trial. Arch. Physiol. Biochem. 126, 1–9. doi: 10.1080/13813455.2020.1856142. PMID: 33370549

[B47] Saeidi A. Tayebi S. M. Khosravi A. Malekian F. Khodamoradi A. Sellam M. . (2019b). Effects of exercise training on type 2-diabetes: the role of Meteorin-like protein. Health Promot. Perspect. 9, 89–91. doi: 10.15171/HPP.2019.12. PMID: 31249794 PMC6588808

[B48] Saxton S. N. Clark B. J. Withers S. B. Eringa E. C. Heagerty A. M. (2019). Mechanistic links between obesity, diabetes, and blood pressure: role of perivascular adipose tissue. Physiol. Rev. 99, 1701–1763. doi: 10.1152/physrev.00034.2018. PMID: 31339053

[B49] Seravalle G. Grassi G. (2016). Obesity and hypertension. High Blood Press Cardiovasc. Prev. 23, 83–90. doi: 10.1007/978-3-031-62491-9_5. PMID: 26942609

[B50] Slivovskaja I. Ryliskyte L. Serpytis P. Navickas R. Badarienė J. Celutkiene J. . (2018). Aerobic training effect on arterial stiffness in metabolic syndrome. Am. J. Med. 131, 148–155. doi: 10.1016/j.amjmed.2017.07.038. PMID: 28864036

[B51] Tayebi S. M. Ghanbari-Niaki A. Saeidi A. Hackney A. C. (2017). Exercise training, Neuregulin 4 and obesity. Ann. Appl. Sport Sci. 5, 1–2. doi: 10.18869/acadpub.aassjournal.5.2.1. PMID: 30899900 PMC6424364

[B52] The Global BMI Mortality Collaboration (2016). Body-mass index and all-cause mortality: individual-participant-data meta-analysis of 239 prospective studies in four continents. Lancet 388, 776–786. doi: 10.1016/S0140-6736(16)30175-1. PMID: 27423262 PMC4995441

[B53] Thijssen D. H. J. Bruno R. M. van Mil A. C. C. M. Holder S. M. Faita F. Greyling A. . (2019). Expert consensus and evidence-based recommendations for the assessment of flow-mediated dilation in humans. Eur. Heart J. doi: 10.1093/eurheartj/ehz350. PMID: 31211361

[B54] Tinken T. M. Thijssen D. H. J. Hopkins N. Dawson E. A. Cable N. T. Green D. J. (2010). Shear stress mediates endothelial adaptations to exercise training in humans. Hypertension 55, 312–318. doi: 10.1161/HYPERTENSIONAHA.109.146282. PMID: 20048193

[B55] Touboul P. J. Hennerici M. G. Meairs S. Adams H. Amarenco P. Bornstein N. . (2012). Mannheim carotid intima-media thickness and plaque consensus (2004-2006-2011): an update. Cerebrovasc Dis. 34, 290–296. doi: 10.1159/000343145. PMID: 23128470 PMC3760791

[B56] Tourny C. Zouita A. El Kababi S. Feuillet L. Saeidi A. Laher I. . (2023). Endometriosis and physical activity: a narrative review. Int. J. Gynaecol. Obstet. 163, 747–756. doi: 10.1002/ijgo.14898. PMID: 37345574

[B57] Townsend R. R. Wilkinson I. B. Schiffrin E. L. Avolio A. P. Chirinos J. A. Cockcroft J. R. . (2015). Recommendations for improving and standardizing vascular research on arterial stiffness: a scientific statement from the American Heart Association. Hypertension 66, 698–722. doi: 10.1161/HYP.0000000000000033. PMID: 26160955 PMC4587661

[B58] Van Craenenbroeck A. H. Van Craenenbroeck E. M. Van Ackeren K. Vrints C. J. Conraads V. M. Verpooten G. A. . (2015). Effect of moderate aerobic exercise training on endothelial function and arterial stiffness in CKD stages 3-4: a randomized controlled trial. Am. J. Kidney Dis. 66, 285–296. doi: 10.1053/j.ajkd.2015.03.015. PMID: 25960303

[B59] Van den Noortgate W. López-López J. A. Marín-Martínez F. Sánchez-Meca J. (2013). Three-level meta-analysis of dependent effect sizes. Behav. Res. Methods 45, 576–594. doi: 10.3758/s13428-012-0261-6. PMID: 23055166

[B60] Van den Noortgate W. Lopez-Lopez J. A. Marin-Martinez F. Sanchez-Meca J. (2015). Meta-analysis of multiple outcomes: a multilevel approach. Behav. Res. Methods 47, 1274–1294. doi: 10.3758/s13428-014-0527-2. PMID: 25361866

[B61] Vehtari A. Gelman A. Gabry J. (2017). Practical Bayesian model evaluation using leave-one-out cross-validation and WAIC. Stat. Comput. 27, 1413–1432. doi: 10.1007/s11222-016-9696-4. PMID: 30311153

[B62] Viechtbauer W. (2010). Conducting meta-analyses in R with the metafor package. J. Stat. Softw 36, 1–48. doi: 10.18637/jss.v036.i03

[B63] Vlachopoulos C. Aznaouridis K. Stefanadis C. (2010). Prediction of cardiovascular events and all-cause mortality with arterial stiffness: a systematic review and meta-analysis. J. Am. Coll. Cardiol. 55, 1318–1327. doi: 10.1016/j.jacc.2009.10.061. PMID: 20338492

[B64] Zouhal H. Rhibi F. Salhi A. Jayavel A. Hackney A. C. Saeidi A. . (2023). The effects of exercise training on plasma volume variations: a systematic review. Int. J. Sports Med. 44, 406–419. doi: 10.1055/a-1667-6624. PMID: 34638157

